# The role of the immunosuppressive PD-1/PD-L1 checkpoint pathway in the aging process and age-related diseases

**DOI:** 10.1007/s00109-024-02444-6

**Published:** 2024-04-11

**Authors:** Antero Salminen

**Affiliations:** Department of Neurology, Institute of Clinical Medicine, University of Eastern Finland, P.O. Box 1627, FI-70211 Kuopio, Finland

**Keywords:** Aging, Immunosuppression, Immune tolerance, Inflammation, Inflammaging

## Abstract

**Abstract:**

The accumulation of senescent cells within tissues is a hallmark of the aging process. Senescent cells are also commonly present in many age-related diseases and in the cancer microenvironment. The escape of abnormal cells from immune surveillance indicates that there is some defect in the function of cytotoxic immune cells, e.g., CD8^+^ T cells and natural killer (NK) cells. Recent studies have revealed that the expression of programmed death-ligand 1 (PD-L1) protein is abundantly increased in senescent cells. An increase in the amount of PD-L1 protein protects senescent cells from clearance by the PD-1 checkpoint receptor in cytotoxic immune cells. In fact, the activation of the PD-1 receptor suppresses the cytotoxic properties of CD8^+^ T and NK cells, promoting a state of immunosenescence. The inhibitory PD-1/PD-L1 checkpoint pathway acts in cooperation with immunosuppressive cells; for example, activation of PD-1 receptor can enhance the differentiation of regulatory T cells (Treg), myeloid-derived suppressor cells (MDSC), and M2 macrophages, whereas the cytokines secreted by immunosuppressive cells stimulate the expression of the immunosuppressive PD-L1 protein. Interestingly, many signaling pathways known to promote cellular senescence and the aging process are crucial stimulators of the expression of PD-L1 protein, e.g., epigenetic regulation, inflammatory mediators, mTOR-related signaling, cGAS-STING pathway, and AhR signaling. It seems that the inhibitory PD-1/PD-L1 immune checkpoint axis has a crucial role in the accumulation of senescent cells and thus it promotes the aging process in tissues. Thus, the blockade of the PD-1/PD-L1 checkpoint signaling might be a potential anti-aging senolytic therapy.

**Key messages:**

- Senescent cells accumulate within tissues during aging and age-related diseases.
- Senescent cells are able to escape immune surveillance by cytotoxic immune cells.
- Expression of programmed death-ligand 1 (PD-L1) markedly increases in senescent cells.
- Age-related signaling stimulates the expression of PD-L1 protein in senescent cells.
- Inhibitory PD-1/PD-L1 checkpoint pathway suppresses clearance of senescent cells.

## Introduction

There are certain traits typically encountered in the aging process which are associated with functional disturbances of the immune system. For instance, a chronic low-grade inflammation, commonly called inflammaging, is a challenge for the maintenance of homeostasis in aged tissues [1]. The low-grade inflammatory state with aging has been linked with an accumulation of pro-inflammatory senescent cells into tissues [2]. The senescence-associated secretory phenotype (SASP) is not only associated with aging but also cells of the tissues with several aged-related diseases display the specific markers of the SASP state [3]. An acute or chronic inflammatory process without a counteracting anti-inflammatory response leads to destructive effects in affected tissues. Thus, there exist diverse anti-inflammatory/immunosuppressive mechanisms which are not only able to suppress inflammatory responses but can also enhance the resolution of the inflammatory state and trigger tissue repair processes. The NF-κB signaling pathway is a major inducer of pro-inflammatory responses [4] and its inhibition, e.g., via endotoxin tolerance [5] or an increased level of the heat-shock proteins HSP70 and HSP90 [6, 7], can suppress inflammatory responses and accordingly enhance immunosuppression. On the other hand, the immune system possesses an armoury of immunosuppressive cells, e.g., myeloid-derived suppressor cells (MDSC), regulatory T cells (Treg), and M2 macrophages, which secrete many immunosuppressive cytokines, such as IL-6, IL-10, IFN-γ, and TGF-β [8–10]. These immunosuppressive mediators act to impair the functions of many immune effector cells and thus promote immunosuppression which subsequently induces the immunosenescence state, i.e., a decline in the activity of the immune system is evident not only in cancer and chronic inflammatory diseases but also in the aging process [11–13].

**Fig. 1:**
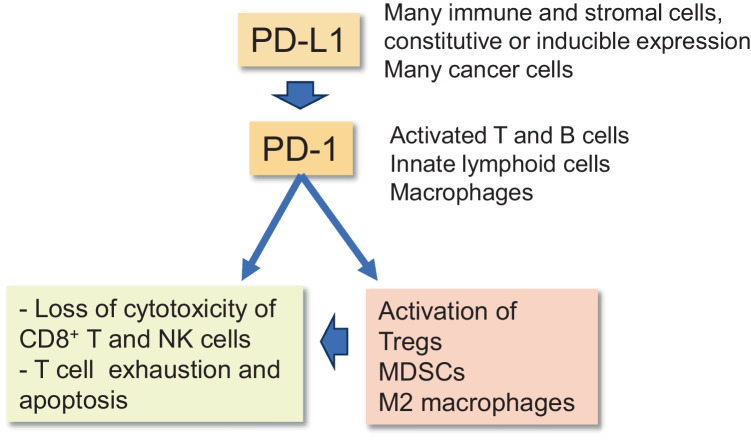
A schematic illustration on the function of PD-1/PD-L1 signaling. The membrane-bound PD-L1 protein activates the PD-1 receptor in a context-dependent manner. The activation of the PD-1 receptor in T, B, or NK cells induces functional disturbances, e.g., a decrease in cytotoxic activity, cellular exhaustion, and apoptosis. PD-1 signaling can also induce the differentiation of immune cells into immunosuppressive Tregs, MDSCs, and M2 macrophages. Moreover, immunosuppressive cells secrete many cytokines which further decrease the activity of the effector immune cells. Abbreviations: MDSC, myeloid-derived suppressor cell; NK, natural killer, PD-1, programmed cell death protein-1; PD-L1, programmed death-ligand 1

Moreover, there are many inhibitory immune checkpoint proteins which provide a crucial mechanism for the maintenance of self-tolerance and the regulation of immune responses, e.g., in chronic inflammatory conditions. For example, the programmed cell death protein-1 (PD-1) and programmed death-ligand 1 (PD-L1) establish an important inhibitory checkpoint axis which has a crucial role in the suppression of T cell activation and thus it can alleviate many immune responses [14] (Fig. 1). In fact, the PD-1/PD-L1 pathway represents a co-inhibitory mechanism which inhibits the activation of the T cell receptor (TCR), thus suppressing antigen presentation via the major histocompatibility complex class II (MHC-II) proteins. Mizuno et al. [15] demonstrated that the binding of the PD-L1 protein to the PD-1 receptor on T cells primarily suppressed the TCR signaling pathway and thus it inhibited cytokine production by mouse CD4^+^ and CD8^+^ T cells. Currently, the inhibitory mechanisms still need to be clarified. Interestingly, the presence of immunosenescence with aging and during age-related diseases is associated with a significant decline in the numbers and functions of CD4^+^ and CD8^+^ T cells as well as in the activity of B, NK, and dendritic cells and tissue macrophages [13, 16, 17]. These age-related changes indicate that the function of the PD-1/PD-L1 signaling pathway might be enhanced during aging and in age-related diseases. In fact, it is known that the expression of the PD-L1 protein is robustly increased in aged tissues and senescent cells [18–20]. This may represent an important mechanism of underpinning both immunosenescence and many age-related immune deficiencies. Here, I will briefly elucidate the properties of the PD-1/PD-L1 checkpoint pathway and then examine in detail the age-related mechanisms promoting the activation of the PD-L1 checkpoint and its role in the aging process and age-related diseases.

## The immunosuppressive PD-1/PD-L1 checkpoint pathway

The inhibitory PD-1/PD-L1 checkpoint pathway confers a significant immunosuppressive protection in autoimmune diseases and many chronic inflammatory states although in cancers and fibrotic diseases it is associated with major detrimental effects [14, 21, 22]. The PD-1 receptor (CD279) is expressed on the surface of the activated T and B cells as well as on macrophages, monocytes, and innate lymphoid cells (ILC) including natural killer (NK) cells [14, 23–25] (Fig. 1). The PD-1 protein is a membrane-bound glycoprotein containing an extracellular IgV domain, a transmembrane sequence, and a cytoplasmic tail which contains a tyrosine-based inhibitory motif (ITIM) and a tyrosine-based switch motif (ITSM). The binding of the PD-L1 protein to the PD-1 receptor induces the phosphorylation of these two inhibitory motifs providing docking sites for the Src-homology-2 (SH2) protein phosphatases. Subsequently, the SHP-1 and SHP-2 phosphatases dephosphorylate the signaling components of the TCR and the costimulatory CD28 pathway, thus suppressing the activation, proliferation, and cytokine production of T cells [23, 26–28]. Stanford et al. [26] have reviewed the function of diverse protein tyrosine phosphatases (PTP) which are involved in the activation and inhibition of the TCR pathway thus regulating the immune homeastasis of T cells. The cytosolic SHP-1, SHP-2, and SHIP1 are major protein phosphatases suppressing TCR signaling associated with the inhibitory function of the PD-1/PD-L1 axis. It is known that PD-1/SHP-1 signaling was able to attenuate the cytotoxic activity of mouse CD8^+^ T cells [29]. Given that NK cells also display PD-1 signaling [25], it seems plausible that cancer cells with their extensive expression of PD-L1 proteins can exploit the PD-1/PD-L1 pathway to suppress the anticancer armoury of the CD4^+^ and CD8^+^ T cells and NK cells, thus escaping the surveillance by the immune system. For instance, Niu et al. [30] demonstrated that the expression of PD-1 protein was significantly increased in the NK cells of human lung cancer. These NK cells exhibited a decline in antitumor activity as compared to the PD-1-negative NK cells. Moreover, it is known that the PD-1 receptor robustly inhibited the activation of human B cells [31]. In 2001, Nishimura et al. [32] demonstrated that the PD-1 receptor-deficient mice developed dilated cardiomyopathy, an autoimmune disorder. Subsequent studies have revealed that many human immunodeficiency diseases are associated with single nucleotide polymorphism (SNP) on human PD-1 gene [33, 34] although the role of the CD-1 receptor in autoimmune diseases still needs to be clarified.

The PD-L1 (CD274 or B7-H1) protein is a type 1 integral membrane glycoprotein and it is expressed in several immune cells, especially in antigen presenting and immunosuppressive cells, as well as in many stromal cells, such as fibroblasts, epithelial and endothelial cells, pericytes, and smooth muscle cells [14, Human Protein Atlas]. There is also an extensive expression of PD-L1 protein in diverse cancer cells and in cancer-associated fibroblasts (CAF), i.e., the PD-1/PD-L1 axis is a major player in the maintenance of the immunosuppressive microenvironment in tumors [35, 36]. Dong et al. [37] cloned the human *B7-H1/CD274* gene (PD-L1) from the human placenta cDNA library. They revealed that the B7-H1/PD-L1 protein did not bind to the CD28 protein, a crucial co-stimulator of T cells, but IL-2 interaction was required to induce T cell proliferation and secretion of immunosuppressive IL-10 cytokine. There also exists the PD-L2 protein (CD273 or B7-DC), mostly expressed in dendritic cells, but the regulation of the PD-1/PD-L2 axis has been less extensively studied than that of the PD-1/PD-L1 counterpart [38]. As discussed above, the PD-L1 protein acts as a ligand which activates PD-1 signaling in T cells and consequently leads to their exhaustion and even apoptosis (Fig. 1). In human NK cells, there are also several other checkpoint proteins, e.g., the HLA-E:CD94-NKG2A complex, which inhibit the immune surveillance by NK cells [39]. It is known that many inflammatory mediators, such as IFNγ, IL-6, IL-10, and TGF-β, are potent inducers of the expression of the PD-L1 protein, e.g., via epigenetic regulation [40, 41]. In inflammatory conditions, the PD-L1-positive cells can activate PD-1 signaling not only through the contact-dependent manner but also by secreting PD-L1-containing extracellular vesicles (EV) which subsequently activate PD-1 signaling in T cells [42]. This EV-mediated signaling is an especially important inducer of immunosuppression in tumors and chronic inflammatory diseases. The regulation of PD-L1 expression will be reviewed more thoroughly in the following sections with respect to cellular senescence and the aging process.

Interestingly, emerging studies have revealed that the PD-L1 protein can also trigger intrinsic effects not only in cancer cells but also in other PD-L1-containing cells, such as in fibroblasts [43–45]. The cell-intrinsic (either cytoplasmic or nuclear) signaling of the PD-L1 protein can regulate many cellular activities, e.g., cellular differentiation, autophagic flux, energy metabolism, and inflammatory responses [45, 46]. For instance, Guo et al. [44] demonstrated that the PD-L1 protein interacted with the Smad3 transcription factor and via β-catenin signaling induced the differentiation of human pulmonary fibroblasts into fibrogenic myofibroblasts. These investigators also reported that the expression of PD-L1 was clearly increased in the lungs of patients with idiopathic pulmonary fibrosis (IPF). It is known that the PD-1/PD-L1 signaling pathway has a crucial profibrotic role in the development of IPF disease [22, 47], although it is not known whether it is caused by intrinsic or extrinsic regulation. Interestingly, Gao et al. [48] demonstrated that the acetylation of the PD-L1 protein at Lys263 within its cytoplasmic tail regulated the translocation of the PD-L1 protein from the cell-surface into the nucleus. The acetylation of the PD-L1 protein by the p300 acetyltransferase blocked its translocation into the nucleus, whereas the deacetylation by histone deacetylase 2 (HDAC2) enhanced its nuclear translocation. The relocation of the PD-L1 protein into the nucleus was driven by clathrin-dependent endocytosis. Gao et al. [48] also revealed results indicating that the nuclear PD-L1 might trigger the expression of those genes which are involved in the immune-response pathways and thus could facilitate the immune evasion of tumor cells. They also reported that the inhibition of the PD-L1 nuclear translocation enhanced the efficacy of the PD-1 blockade therapy.

**Fig. 2:**
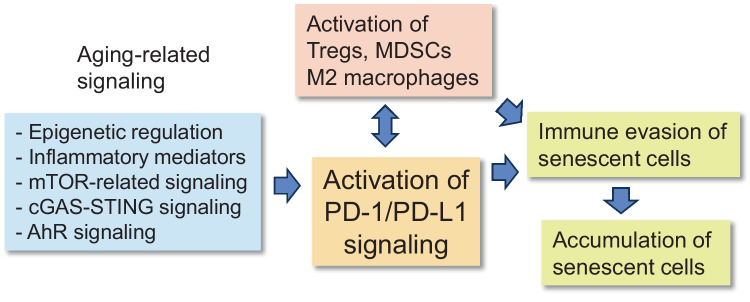
The role of PD-1/PD-L1 signaling in the accumulation of senescent cells with aging. Several aging-associated signaling pathways can stimulate the expression of PD-L1 protein which via PD-1/PD-L1 signaling suppresses immune surveillance of senescent cells and thus promotes their accumulation within aging tissues. The collaboration with immunosuppressive cells also enhances immunosenescence in aged tissues and thus helps senescent cells to evade immune surveillance and subsequent elimination. Abbreviations: AhR, aryl hydrocarbon receptor; cGAS-STING, cyclic GMP-AMP synthase stimulator of interferon response cGAMP interactor; mTOR, mechanistic target of rapamycin

The PD-1/PD-L1 signaling pathway is also able to enhance the differentiation of immune cells towards the immunosuppressive state (Fig. 1). For instance, there is robust evidence that an increase in PD-1 signaling can promote the differentiation of CD4^+^ T cells into immunosuppressive Treg cells in diverse experimental models [49, 50]. It seems that cancer chemotherapy with the anti-PD-L1 antibody can alleviate both the inhibition of T cells and suppress the differentiation of Tregs. Interestingly, the expression level of PD-1 receptor controls the polarization of macrophages, i.e., a deficiency of PD-1 expression promoted the development of the pro-inflammatory M1 phenotype, whereas its overexpression enhanced the appearance of the immunosuppressive M2 phenotype, especially in tumor-associated macrophages (TAM) [51, 52]. Wei et al. [52] reported that the M2 polarization was induced via the activation of the ERK/AKT/mTOR signaling pathway in human macrophages. TAMs have an important role in the maintenance of an immunosuppressive state in the tumor microenvironment. In addition, it is known that PD-1 signaling enhanced the proliferation and immunosuppressive activity of MDSC, especially in the microenvironment of tumors [53] and sepsis [54]. It seems that the PD-L1-induced activation of PD-1 enhances immunosuppression either by suppressing effector immune cells, such as T, B, and NK cells, or by stimulating the differentiation of immunosuppressive Tregs, MDSCs, and M2 macrophages (Fig. 1). On the other hand, immunosuppressive cells secrete many cytokines, such as IL-6, IL-10 and TGF-β [55, 56], which are potent inducers of the expression of PD-L1, i.e., the PD-1/PD-L1 checkpoint axis acts in cooperation with immunosuppressive cells (Fig. 2).

## The aging process upregulates the expression of the PD-L1 protein

### Aging tissues

There is abundant evidence that the aging process is associated with the activation of an immunosuppressive network [10, 57]. The numbers of MDSCs, Tregs, and M2 macrophages increase not only in the secondary lymphoid organs but also in many peripheral tissues [58–62]. However, less is known about the expression of PD-1/PD-L1 components in aging tissues although it represents a potent immunosuppressive mechanism in many pathological conditions. Several investigators have reported that the expression levels of PD-1 and PD-L1 proteins were robustly increased in many tissues with aging, e.g., bone marrow, heart, kidney, liver, lung, cerebellum, and spleen [18–20, 63, 64]. Currently, the cellular source of the age-related increase in the expression of PD-L1 protein needs to be clarified in different tissues although Yousefzadeh et al. [2] reported that the expression levels of the p16 and p21, markers of cellular senescence, were significantly increased in the brain, heart, kidney, liver, lung, and spleen in aged mice.

### Cellular senescence

There are several hallmarks of cellular senescence, e.g., irreversible cell-cycle arrest, impaired proteostasis and energy metabolism, and the SASP state with the secretion of many inflammatory mediators, such as cytokines and chemokines [65]. Interestingly, senescent cells also secrete exosomal vesicles which have immunosuppressive properties, e.g., carrying the membrane-bound PD-L1 proteins [66]. In their seminal study, Onorati et al. [18] demonstrated that the expression of PD-L1 protein was robustly upregulated in human IMR90 and BJ fibroblasts after the cells were exposed to different inducers of cellular senescence. They also reported that the induction of PD-L1 expression was mediated by secreted inflammatory factors via the activation of the JAK/STAT3 signaling pathway. Accordingly, Wang et al. [20] demonstrated that PD-L1-positive senescent cells accumulated into mouse lung, liver, and kidney with aging. Single-cell transcriptome analysis revealed that the expression of PD-L1 correlated with an increased level of inflammatory factors in senescent cells.

Given that the accumulation of senescent cells promotes the aging process, it is clearly important to clarify whether treatment with an anti-PD-L1 antibody would be able to enhance the elimination of senescent cells in aging tissues. Wang et al. [20] demonstrated in vitro that those mouse pulmonary fibroblats which expressed a high level of PD-L1 protein were more resistant to the surveillance of cytotoxic CD8^+^ T cells than their PD-L1-negative counterparts. This confirms that the PD-L1-positive senescent fibroblasts were able to suppress the activity of cytotoxic PD-1-positive T cells and thus evaded immune clearance. Wang et al. [20] also revealed that the treatment of aging mice with an anti-PD-1 antibody reduced the numbers of both senescent cells and PD-L1-positive cells in mouse liver indicating that senescent cells were not able to evade immune elimination after immunotherapy.

On the other hand, it is known that the expression of PD-1 protein increases in senescent human T cells and thus it exposes them to the exhaustion/apoptosis by the PD-L1-positive stromal/immune cells [67, 68]. For instance, Kasamatsu et al. [68] demonstrated that sublethal doses of chemotherapeutic agents induced a senescent phenotype in human T cells and significantly increased the expression of PD-1 protein in these cells. Interestingly, it seems that with aging or in age-related diseases, senescent stromal cells possess a dual defence against immune elimination, i.e., an increased expression of PD-L1 protein improves their immunodefence against cytotoxic immune cells and moreover, the senescence/exhaustion of T cells with aging increase their expression of PD-1 protein which exposes them to the elimination by PD-L1-positive senescent stromal cells. Currently, it seems that in cancer therapies there are no significant differences between young adults and elderly people in the efficacy of the blocking treatments utilizing PD-L1 and PD-1 antibodies [69].

## PD-1/PD-L1 signaling is augmented in many age-related diseases

The age-related diseases are progressive disorders which commonly involve both inflammatory changes and fibrotic degeneration. Typically, these diseases also are associated with the accumulation of senescent cells with inflammatory and degenerative changes, such as in atherosclerosis, chronic obstructive lung disease (COPD), coronary artery disease (CAD), and Alzheimer’s disease (AD) [70–73] as well as in fibrotic diseases, e.g., in idiopathic pulmonary disease (IPF), cardiac fibrosis, and systemic sclerosis [70, 74, 75]. There exists a significant crosstalk between senescent cells and inflammatory cells in the pathogenesis of many age-related diseases, e.g., via the control of the expression of inhibitory checkpoint proteins. For instance, the PD-1/PD-L1 axis has an important role in the pathogenesis of human AD and COPD [76–78]. Kummer et al. [79] demonstrated that the expression of PD-L1 was robustly increased in the brain of transgenic AD mice with clear positive staining in the astrocytes surrounding amyloid-β plaques. Moreover, a deficiency of microglial PD-1 protein increased the inflammatory response and the deposition of amyloid-β plaques within the brain. Given that inflammatory mediators are potent inducers of the expression of PD-L1 protein, it seems that in many age-related diseases, the activation of PD-1/PD-L1 signaling is related to defence against chronic inflammation. The pro-inflammatory SASP state of senescent stromal cells maintains the inflammatory microenvironment and aggravates the pathology of age-related diseases. It seems plausible that the induction of PD-L1 expression in senescent cells has a crucial role in the pathogenesis of chronic age-related diseases since it promotes immunosenescence and prevents the clearance of senescent cells.

Fibrosis in cardiac muscle, kidney, liver, and lung as well as in cases of systemic sclerosis are common age-related fibrotic diseases. The differentiation of tissue fibroblasts into fibrogenic myofibroblasts and an increase in the numbers of senescent fibroblasts are the major hallmarks of fibrotic lesions [75, 80]. Emerging studies have revealed that the function of the PD-1/PD-L1 axis has a crucial role in the accumulation of fibrotic lesions [22, 47]. There are a number of investigations indicating that in mouse and human fibrotic tissues, there exists a robust increase in the expression of PD-L1 protein not only in fibroblasts but also in endothelial and epithelial cells [18, 22, 44, 47, 81]. Onorati et al. [18] utilized a single-cell transcriptome technique to determine in human interstitial lung fibrosis that the high expression of PD-L1 protein was localized into senescent cells which also displayed a proinflammatory phenotype. There are studies indicating that the PD-L1 protein might promote fibrosis by enhancing the differentiation of tissue fibroblasts into fibrogenic myofibroblasts. For example, Guo et al. [44] demonstrated that the PD-L1 protein was required for the TGF-β-induced differentiation of myofibroblasts both from human primary lung fibroblasts or fibroblasts isolated from the IPF patients. They revealed that TGF-β exposure induced the interaction between the PD-L1 protein and the SMAD3 transcription factor to induce the transcription of α-smooth muscle actin (α-SMA). It seems that the PD-L1 protein can enhance tissue fibrosis via some form of intrinsic regulation. There are several reports that immunosuppressive CAFs express a high level of PD-L1 protein [82, 83]. For instance, Li et al. [82] demonstrated that CAFs were able to promote the expression of PD-L1 protein in mouse cancer cells by secreting the CXCL5 chemokine. Given that the inhibitors of PD-1/PD-L1 signaling are effective in many cancers, the checkpoint inhibitors may also represent promising therapeutic choices for treating fibrotic diseases [84]. Currently, there are some animal studies in experimental disease models which have revealed that the PD-1/PD-L1 inhibitors are able to attenuate the severity of the fibrosis [85, 86].

## The aging-associated signaling stimulates the expression of the PD-L1 protein

Given that the PD-1/PD-L1 pathway has an important role in the generation of an immunosuppressive microenvironment in tumors, there has been a significant interest in examining the regulation mechanisms underpinning the expression of the PD-L1 protein [43, 87, 88]. It seems that there exists a complex network of regulatory processes from epigenetic to post-transcriptional mechanisms which not only control the expression level of PD-L1 but also the resistance to the PD-1/PD-L1 blocking therapies. Currently, the signaling mechanisms which regulate the age-related increase in the expression of PD-L1 still need to be clarified although many of them are known to be associated with the regulation of the aging process. Next, I will briefly examine some of those signaling pathways and emphasize that the upregulation of the PD-1/PD-L1 pathway seems to be involved in the regulation of the aging process (Fig. 2).

### Epigenetic regulation

There is robust evidence from cancer studies that different forms of epigenetic regulation, such as DNA methylation and histone modifications, control the chromatin landscape around the *PD-L1* gene and affect its transcription [89, 90]. For instance, the *PD-L1* gene contains many CpG methylation sites on its 5’-untranslated region as well as on exon 1. The hypomethylation of the CpG clusters on the promoter regions of the *PD-L1* gene is a major hallmark encountered in different tumors, e.g., in melanoma and colorectal cancer [91, 92]. It is generally recognized that a high methylation level of the *PD-L1* promoter is associated with a low expression of PD-L1. For example, exposure to 5-azacytidine, an inhibitor of DNA methyltransferase, increased the degree of hypomethylation and elevated the expression of the *PD-L1* gene [93]. However, there are reports indicating that certain CpG loci in the *PD-L1* promoter were actually hypermethylated in some cancers, e.g., in advanced gastric cancer [94], thus it seems that not all methylation sites are involved in the regulation of PD-L1. It is not only in cancers where it is believed that DNA methylation controls the expression of the *PD-L1* gene. For instance, Chang et al. [95] demonstrated that the demethylation of the *PD-L1* promoter enhanced the expression of PD-L1 and consequently inhibited the experimental autoimmune encephalomyelitis in mice.

The regulation of histone acetylation and deacetylation is another epigenetic mechanism which regulates the transcription of the *PD-L1* gene and thus it is an important therapeutic target [96]. The acetylation of histones facilitates the opening of chromatin and consequently enhances the transcription of genes. Histone acetyltransferases (HAT) increases the acetylation of histones, whereas HDACs remove acetyl groups from histones, thus decreasing transcription. Wang et al. [97] demonstrated that the activation of JNK/c-Jun signaling stimulated the expression of PD-L1 in diverse drug-resistant cancer cell lines. Subsequently, they revealed that the accumulation of c-Jun protein inhibited the expression of HDAC3 and thereby increased the acetylation of histone H3 on the PD-L1 promoter in cancer cells. Accordingly, the overexpression of HDAC3 reduced H3 acetylation and inhibited the expression of PD-L1. There are many studies indicating that the inhibition of HDACs increased the expression of PD-L1 protein in human and murine cancer cells and consequently augmented the efficacy of anti-PD-L1 therapy, e.g., in B-cell lymphomas [98] and mouse experimental tumor models [99]. Currently, certain HDAC inhibitors are clinically used as anticancer agents. Moreover, microRNAs (miR) are important players which can control the expression level of PD-L1 [100] and subsequently inhibit tissue immunosuppression. Wang et al. [100] categorised a number of miRs which can directly or indirectly inhibit the expression of PD-L1.

The remodelling of the chromatin landscape is a hallmark of both cellular senescence and the aging process [101–103]. Currently, there is some debate whether this represents a cause or consequence of the aging process. Shortly, the following changes have been observed, (i) an increase in global DNA hypomethylation although certain CpG islands may be hypermethylated, (ii) a reduction in the level of heterochromatin and nucleosomes, and (iii) clear changes in histone structure and epigenetic marks. Benayoun et al. [64] demonstrated that the age-related increase in an inflammatory phenotype was associated with both epigenetic remodelling and changes in tissue transcriptome profiles. For instance, many genes of the immune pathways displayed an enrichment of the activating epigenetic marks, such as greater intensities of the H3K4me3 and H3K27ac, and accordingly an increase in their mRNA expression. Interestingly, the IFNγ response, a major inducer of PD-L1 transcription [40], displayed an age-related increase in activating epigenetic marks around the gene as well as an increased transcription in mouse heart, liver, and cerebellum. Erbe et al. [104] surveyed the multi-omics databases focusing on the age-related transcription of immune checkpoint genes, also that of the *PD-L1* gene. They reported that an increased transcription of the *PD-L1* gene was associated with a clear decrease in the degree of promoter methylation. Currently, the aging-associated changes in different CpG loci in the promoter of the *PD-L1* gene need to be clarified. However, it seems that an age-related increase in the expression of PD-L1 and accordingly the enhanced tissue immunosuppression can be partly attributed to the global DNA hypomethylation that takes place with aging.

#### Inflammatory mediators

The aging process is associated with an accumulation of senescent cells as well as an increase in pro-inflammatory changes, not only in the organs of immune system but also in peripheral tissues. As described above, Benayoun et al. [64] demonstrated that the age-related inflammatory signature in many tissues was associated with epigenetically driven alterations in the transcription of inflammatory genes involving cytokines, chemokines, complements, and the response to interferons. Moreover, the age-related changes were very similar in the tissues of mice, rats, and humans. Interestingly, recent single-cell transcriptome experiments on aged tissues have revealed that the inflammatory response is induced not only by infiltrating immune cells but that there is also a robust increase in the markers of the inflammatory phenotype in the stromal cells of the tissues, such as fibroblasts, endothelial, and epithelial cells [105–108]. Given that most of the inflammatory markers are probably derived from senescent cells, many single-cell transcriptome screening trials have examined the coordination of different type of senescent genes (SnG), e.g., cell-cycle inhibitors and the SASP genes, in aging tissues of human and mice [109, 110]. For instance, Xu et al. [110] reported that many SnGs were enriched in distinct clusters in several human tissues. Fibroblasts were the most prominent senescent cell type across the tissues although several other stromal cells displayed many properties of senescence. In particular, diverse cytokine regulators, e.g., the interferon regulator STAT3 and the cytokine regulator RelA from the NF-κB pathway, were enriched with common cell cycle inhibitors in the examined aging tissues. Currently, the high-throughput screening and multiomic studies have tended to focus on assessing the common age-related alterations rather than examining more specific immune pathways.

Currently, it is known that a diverse set of inflammatory mediators, associated both with acute and chronic inflammatory conditions, are able to stimulate the expression of PD-L1. However, it seems that the NF-κB and JAK/STAT pathways are the major inflammation-related inducers of transcription of the *PD-L1* gene [21, 40, 111–113]. For instance, many studies have revealed that IFN-γ is a major inducer of PD-L1 and PD-L2 expression via the JAK/STAT pathways in several cancer models [40, 111]. Garcia-Diaz et al. [111] demonstrated that the STAT1/3 signaling targeted the interferon regulatory factor 1 (IRF1) which subsequently transactivated the promoter of the *PD-L1* gene in human melanoma cells. Not only cytokines, such as IL-1β, IL-17, TNF-α, and GM-CSF, but also reactive oxygen species (ROS), UV irradiation, and lipopolysaccharide triggered the expression of PD-L1 via NF-κB signaling under many experimental conditions [114–119]. Recently, Lu et al. [120] revealed that the NLRP3 inflammasome, which is primed by NF-κB signaling, upregulated the expression of PD-L1 in mouse lymphoma. They also reported that a blockade of NLRP3 signaling (i) inhibited the expression of PD-L1, (ii) reduced the level of immunosuppressive cells, and (iii) subsequently blocked the growth of mouse lymphoma. Moreover, it is known that the non-canonical RelB pathway of NF-κB signaling increased the expression of PD-L1 protein and allowed mouse prostate cancer cells to evade surveillance by the immune system [121]. The complement system might also be involved in the regulation of PD-L1 expression. For example, An et al. [122] demonstrated that treatment of human monocytes with the complement factor C5a induced the expression of PD-L1 protein which was mediated via the activation of the complement receptor C5aR1. Accordingly, Zha et al. [123] reported that the inhibition of C5aR signaling promoted the efficiency of PD-1/PD-L1 inhibitor therapies in mouse tumors. These studies indicate that diverse pro-inflammatory treatments are able to stimulate the expression of PD-L1 and thus evoke tissue immunosuppression through the PD-1/PD-L1 pathway.

It is known that TGF-β and prostaglandin E2 (PGE2) are important immunosuppressive factors and enhancers of immune suppressor cells, such as MDSCs and M2 macrophages. Interestingly, there are studies indicating that PGE2 and TGF-β are also potent inducers of PD-L1 expression [124, 125]. For example, Kang et al. [125] demonstrated that TGF-β exposure not only stimulated the expression of PD-L1 in human fibroblasts but it also triggered an exosomal secretion of PD-L1 proteins. They also reported that TGF-β treatment induced the transactivation of the *PD-L1* gene either via the SMAD2/3 pathway or through YAP/TAZ signaling. There are clinical observations indicating that the simultaneous inhibition of TGF-β and PD-1/PD-L1 signaling conferred synergistic benefits in cancer therapy [126]. Prima et al. [124] revealed that PGE exposure increased the expression level of PD-L1 in mouse macrophages and accordingly enhanced their immunosuppressive activity in tumor microenvironment. Interestingly, it is known that the synthesis of PGE2 was significantly upregulated in the macrophages isolated from aged mice, a phenotype which was attributable to an increase in the expression of cyclo-oxygenase 2 (COX-2) [127]. Moreover, Li et al. [128] demonstrated that the expression levels of COX-2 and PGE synthase 1 (PTGES1) were robustly augmented in the skin fibroblasts of aged humans, concurrently with an increased concentration of PGE2. These observations might be connected to the accumulation of senescent cells and the immunosenescence found in the aged skin. In summary, there is abundant evidence that many pro-inflammatory factors stimulate the expression of PD-L1 protein and that this property can even be promoted by immunosuppressive mediators, such as PGE2 and TGF-β.

### mTOR-related signaling

The mechanistic target of rapamycin (mTOR) is a key protein kinase which regulates a number of vital cellular functions involving, e.g., cell growth and survival, protein synthesis, protein degradation via autophagy, metabolism, and many immune responses [129, 130]. Thus, it is generally believed that mTOR signaling is a major regulator of the aging process and many age-related diseases [131]. It is known that (i) mTOR signaling inhibits autophagy and (ii) autophagic activity declines with aging, i.e., cleansing of cellular components is reduced with aging which disturbs the maintenance of homeostasis [132, 133]. However, currently this paradigm contains many open questions since both mTOR signaling and autophagy are heavily regulated upstream and furthermore they are driving many diverse functions which seem to be cell-type specific. With respect to the upstream regulation, many growth factors, such as insulin and IGF-1, and many cytokines are connected to the PI-3 K/AKT/mTOR signaling pathway which can be inhibited by the PTEN protein phosphatase. This pathway is a key regulator of mTOR-driven immune responses, such as the activation and differentiation of T and B cells as well as the antigen presenting cells [130] and moreover, mTOR signaling has a crucial role in the development of immune evasion in the tumor microenvironment [134]. In addition, there exist two kinds of autophagy, i.e., a nonselective bulk macroautophagy and a chaperone-assisted selective autophagy for specific targets ranging from distinct molecules to whole organelles [135]. For instance, it is known that selective autophagy controls many innate immune responses [136].

There is evidence emerging from different experimental models that the inhibition of autophagy by mTOR signaling can stimulate the expression level of PD-L1 protein [137–139]. The early observations indicated that the activation of the PI-3 K/AKT/mTOR signaling pathway induced the expression of PD-L1. For instance, Parsa et al. [140] demonstrated that the activation of the PI-3 K/AKT/mTOR pathway by inhibiting the PTEN phosphatase stimulated the expression of PD-L1 protein in glioma cells. They also revealed that the level of PD-L1 protein increased through translational regulation and consequently enhanced the extent of immunoresistance in an experimental mouse glioma. Subsequently, Wang et al. [137] made a seminal observation indicating that the inhibition of autophagy, either with pharmacological inhibitors or after knockout of autophagic genes, robustly increased the expression of PD-L1 in mouse and human gastric cancer cells. They also reported that the induction of autophagy by rapamycin, an inhibitor of mTOR, reduced the expression levels of PD-L1 in cancer cells. This indicates that mTOR signaling inhibits autophagy and consequently augments the expression of PD-L1 protein and conversely the activation of autophagy decreases the level of PD-L1 protein. Wang et al. [137] also demonstrated that the inhibition of autophagy induced the accumulation of p62/SQSTM1 protein, a common receptor of selective autophagy [141], indicating that the PD-L1 protein is degraded by a process of selective autophagy. This is an interesting observation since a decline in the autophagic flux with aging might explain why the aging process is associated with an increase in the expression of PD-L1.

For many years, it has been known that the PD-L1 protein also possesses many cell-intrinsic, nonimmune functions [43, 142]. For example, Gao et al. [143] demonstrated that the PD-L1 protein activated the AKT/mTOR signaling pathway and increased the proliferation of human ovarian cancer cells. Accordingly, Clark et al. [144] reported that the PD-L1 protein mediated a suppression of autophagy via a cell-intrinsic, immune-independent pathway in murine ovarian cancer and melanoma cells. They revealed that the PD-L1 of tumor cells promoted mTOR signaling which inhibited autophagic flux and thus maintained the expression of PD-L1 protein upregulated in tumor cells. However, it seems that there are some cell-type dependent regulatory differences in this bidirectional interplay between the PD-L1 protein and the phenomenon of autophagy [43, 142].

### cGAS-STING signaling

The cyclic GMP-AMP synthase (cGAS)-stimulator of interferon genes (STING) is a cytoplasmic sensor for double-stranded DNA breaks which can originate from either nuclear, mitochondrial, or microbial DNA [145, 146]. The primary cause of aging is still unknown although many investigators have suggested that a loss of DNA integrity, e.g., genome instability, telomere attrition, and dysfunctions in mitochondrial DNA (mtDNA), is a hallmark of the aging process [147]. There is robust evidence that the activation of cGAS-STING signaling is associated with cellular senescence and inflammatory states driven by DNA damage [148–150]. Recently, Gulen et al. [151] demonstrated that cGAS-STING signaling promoted both age-related inflammation and neurodegeneration in mice. They also reported that the inhibition of STING activity blocked the inflammatory SASP response in cultured senescent human fibroblasts. Next, they revealed that if old mice were administered an inhibitor of STING activity, this significantly attenuated the inflammatory signature in their brain, kidney, and liver. Gulen et al. [151] demonstrated that the levels of the phosphorylated STING, a marker of its activation, were enriched in the hippocampus of aged mice, especially in microglia. They also reported that mtDNA triggered the activation of cGAS-STING signaling in aged microglia and thus induced an inflammatory state in the aged mouse brain.

It is known that STING signaling stimulates the activation of IRF3 and NF-κB signaling pathways as well as promoting autophagic degradation [145, 146]. There is clear evidence from many clinical experiments that the activation of STING (i) induced the expression of PD-L1 protein, (ii) inhibited T cell function, and (iii) promoted immune evasion in many clinical experiments [152, 153]. For instance, Cheng et al. [152] demonstrated that oxidative stress in cancer cells activated the mitochondrial Lon protease, subsequently promoting the release of mtDNA into the cytoplasm where it activated cGAS-STING signaling and stimulated the expression of PD-L1 protein. Interestingly, they revealed that an increased Lon activity in M2 macrophages promoted the secretion of EVs which carried a cargo of mtDNA and the PD-L1 protein capable of inhibiting the function of T cells. Moreover, it is known that ionizing radiation therapy triggered the activation of cGAS-STING signaling and induced the upregulation of PD-L1 expression in the cell-surface of human and mouse liver cancer cells and subsequently it enhanced immune evasion in mouse tumor model [153]. On the other hand, Lee et al. [154] demonstrated that via its intrinsic regulation, the nuclear PD-L1 protein inhibited the transcription of STING protein by binding to the promoter region of the *STING* gene in cancer cells. Accordingly, silencing of PD-L1 expression induced a robust upregulation of STING expression and subsequently inducing cellular senescence of mouse cancer cells. It seems that there is a negative feedback regulation between the cGAS-STING and PD-L1 signaling pathways and an increase in PD-L1 expression with aging might inhibit autophagy and aggravate the maintenance of genome integrity.

### AhR signaling

The aryl hydrocarbon receptor (AhR) is an evolutionarily conserved, ligand-regulated transcription factor which is not only a major sensor of environmental toxins but it is also activated by a number of metabolites ranging from heme, arachidonic acid, and kynurenine pathways as well as indoles from microbiota [155, 156]. AhR signaling cooperates closely with indoleamine 2,3-dioxygenase 1 (IDO1), a common immune checkpoint, in the regulation of the aging process [157, 158]. For instance, AhR signaling can remodel the immune system by stimulating immunosuppressive changes, e.g., through the induction of FoxP3 and the differentiation of Tregs [157, 159]. Interestingly, the promoter of the *PD-L1* gene contains several response elements capable of binding the AhR factor (AhRE) [160]. Kenison et al. [160] demonstrated that the activation of AhR signaling by TCDD exposure transactivated the *PD-L1* gene in cancer cells, whereas the AhR knockout robustly reduced the induction of the *PD-L1* gene. It has been demonstrated in many experimental models that AhR signaling can stimulate the expression of PD-L1 protein [161–163]. For example, Wang et al. [161] revealed that cigarette smoke, a potent activator of AhR signaling, induced in vivo the expression of PD-L1 protein in the epithelial cells of mouse lungs. They also verified that the induction of PD-L1 was caused by AhR signaling. Moreover, Wang et al. [161] reported that the inhibition of AhR signaling potentiated the therapeutic response induced with anti-PD-L1 antibody treatment in mouse lung cancer model. Tryptophan metabolites, e.g., compounds produced via the kynurenine, indole, serotoinin, or tryptamine pathways, are activators of AhR signaling as well as immune modulators [156, 164]. Currently, there is clear evidence that the gut microbiota can modulate the efficacy of anti-PD-1/PD-L1 therapy both in humans and mice [165]. The gut microbiota generates several indole derivatives which are potent agonists of the AhR protein in many tissues via the circulation [156]. For example, oxidative stress triggered the formation of 2-oxindole which stimulated the expression of PD-L1 in human keratinocytes [162]. It does seem that the robust expression of the AhR protein in the barrier tissues, such as vascular tissues, has an important role in the regulation of the aging-associated immunosuppression and immunosenescence [157].

## The anti-aging signaling suppresses the expression of the PD-L1 protein

Currently, there is a debate if it will be possible to develop anti-aging therapies which might increase health span and even extend the lifespan of elderly humans. Recently, Guarente et al. [166] summarized the present clinical knowledge and ongoing trials on the eight most promising drug candidates for anti-aging medicines. Two of these drugs, i.e., metformin and rapamycin, are clinically utilized drugs which have been able to extend the lifespan of rodents [167, 168]. Metformin, a drug used in the clinical treatments of type 2 diabetes, has multiple targets in the body, e.g., it activates the AMP-activated kinase (AMPK) which controls many aging-associated signaling pathways [169]. Interestingly, metformin has displayed diverse therapeutic properties in many immune-mediated diseases [170] and also alleviated many of the hallmarks of cancers [171]. For instance, metformin exposure inhibited the function of immunosuppressive MDSCs in tumor-bearing mice [172]. This is probably induced by the activation of AMPK signaling which is known to inhibit the functions of MDSCs [173]. There is abundant evidence that metformin can reduce the expression of PD-L1 protein through diverse mechanisms, although mainly via AMPK signaling [174–176]. Cha et al. [174] demonstrated that metformin via AMPK activation phosphorylated the PD-L1 protein at the Ser195 site in cancer cells. The phosphorylation of the PD-L1 protein induced its glycosylation and consequently proteasomal degradation via the endoplasmic-reticulum-associated degradation (ERAD) pathway. Cha et al. [174] also reported that the metformin-induced downregulation of the amount of PD-L1 protein present in cancer cells enhanced their surveillance by cytotoxic T lymphocytes. They also verified that metformin exposure reduced the level of PD-L1 in human tumors. It seems that there are other mechanisms which metformin can exploit to inhibit the expression of PD-L1 protein. Lu et al. [175] revealed that metformin treatment inhibited the expression of PD-L1 by suppressing the IL-6/JAK2/STAT3 pathway in human esophageal squamous carcinoma cells. This inhibition was an AMPK-independent process. However, it seems likely that many metformin-induced responses are mediated by AMPK-induced autophagy, either by inhibiting mTOR or activating ULK1 signaling [177].

Rapamycin, an inhibitor of mTOR signaling, is a potent enhancer of autophagy. As discussed above in the section ‘mTOR-related signaling’, the signaling pathways via the activation of mTOR stimulate the expression of PD-L1 protein in many experimental conditions. Given that rapamycin has been able to extend the lifespan of rodents [167], the inhibitors of mTOR protein, such as rapalogs, have been claimed to be promising candidates for anti-aging therapeutics [178]. Khedri et al. [179] demonstrated that exposure of mice to rapamycin significantly reduced the expression of PD-L1 in the brain but not in the spleen. They proposed that rapamycin might have beneficial effects on CNS autoimmune diseases, such as those encountered in multiple sclerosis [180]. Onorati et al. [18] reported that rapamycin exposure was able to downregulate the expression of PD-L1 protein, especially on the cell surface of senescent human fibroblasts. Rapamycin treatment also reduced the expression of IL-6 in senescent cells, an effect associated with a decline in the SASP state. This is an interesting observation since a decrease in the expression of PD-L1 in senescent cells might enhance their immune surveillance and thus rapamycin could possess senolytic properties.

Traditional medicine and phytochemicals have long been exploited in health care for diverse diseases and even as anti-aging remedies. Many clinically used drugs, such as aspirin and metformin, have their roots in herbal medicine. Phytochemicals, mostly polyphenols, are multitarget molecules, like metformin, and they affect many age-related signaling pathways. In fact, it is known that several phytochemicals possess immunomodulatory properties [181]. For instance, there is clear evidence that phytochemicals can improve the efficacy of many cancer therapies based on PD-1/PD-L1 blockade [182, 183]. Currently, the molecular mechanisms need to be clarified before phytochemicals can be administered as checkpoint inhibitor therapies. Guo et al. [184] revealed in in silico experiments that several polyphenols, such as curcumin and resveratrol, were able directly to inhibit the interaction between the PD-1 and PD-L1 proteins. Accordingly, Jing et al. [185] reported that quercetin also inhibited the interaction between the PD-1/PD-L1 proteins and thus promoted the ability of T cells to destroy human cancer cells. Phytochemicals can also enhance the efficacy of immunotherapy with PD-1/PD-L1 inhibitors by downregulating the expression of PD-L1 in cancer cells. For instance, Liu et al. [186] demonstrated that curcumin exposure suppressed the expression of PD-L1 and PD-L2 proteins in human head and neck squamous carcinoma cells (HNSCC). They also reported that curcumin potentiated the cytotoxic activity of CD8^+^ T cells when administered as a combination treatment with PD-L1 antibody. Recently, Liu et al. [187] reviewed the molecular mechanisms describing how different phytochemicals alter the function of the PD-1/PD-L1 pathway in tumor immunotherapy.

## The PD-1/PD-L1 checkpoint signaling promotes aging-related immunosuppression and immunosenescence

There is convincing evidence that the expression of PD-L1 protein is significantly increased in many tissues with aging. This age-related response seems to be attributable to an accumulation of senescent cells which are known to exhibit a robust expression of the PD-L1 protein. This increased expression level of the PD-L1 protein indicates that senescent cells can evade immune clearance by inhibiting the activity of cytotoxic CD8^+^ T and NK cells. This might explain why senescent cells accumulate with aging and disturb the maintenance of tissue homeostasis. Senescent cells not only express the PD-L1 protein but they can also secrete EVs coated with PD-L1 proteins. It is known that the shedding of EVs is robustly increased from the senescent cells of diverse cell types, e.g., fibroblasts and endothelial cells [188, 189]. The secretion of EVs enhances the paracrine spreading of senescence into neighboring cells within tissues [190, 191]. Borghesan et al. [190] reported that the IFN-γ inducible transmembrane 3 protein (IFITM3) was able to increase the secretion of EVs and thus promote the paracrine senescence in aged tissues. Interestingly, Benayoun et al. [64] demonstrated that in mice, the gene transcription of the IFN-γ-driven genes was strongly enriched in different aging tissues. Moreover, Kang et al. [125] demonstrated that TGF-β exposure of human and mouse fibroblasts also induced the secretion of the PD-L1-coating EVs which inhibited the function of T cells. There is mounting evidence that the PD-L1-coated EVs, secreted either from cancer cells or CAFs, have a key role in the maintenance of immunosuppressive state in the microenvironment surrounding tumors [192, 193]. It is known that chronic inflammatory conditions also increase the secretion of immunosuppressive EVs [66]. It seems that an excessive expression of PD-L1 protein and their packaging into EVs in senescent cells not only induces an accumulation of senescent cells into aged tissues but also promotes the generation of immunosenescence with aging by inhibiting the functions of T and NK cells (Fig. 2).

The immunosenescence associated with aging involves a substantial decline in the functional properties of the immune system [13, 16, 194]. Furthermore, the immunosenescence present in tumors displays more or less similar alterations in immune cells as those encountered in the aging process [11]. Currently, it is known that the PD-1/PD-L1 checkpoint axis impairs the functions of T and B cells as well as NK cells and macrophages, inducing relatively similar changes as those observed in immunosenescence, i.e., an activation of the PD-1/PD-L1 pathway with aging might promote the aging of the immune system. However, it needs to be clarified whether other inhibitory immune checkpoints, such as CTLA-4, LAG-3, TIM-3, or VISTA, affect the age-related immune deficiency. In addition to the contact-mediated inhibition, immunosuppressive cells, such as MDSCs, Tregs, and M2 macrophages, secrete cytokines which evoke a decline in the functions of both adaptive and innate immunity [9, 13, 55, 58]. Interestingly, immunosuppressive cells are able to induce alterations in immune cells which are very reminiscent to those observed in the immunosenescent phenotype [13, 195]. In fact, it is known that the contact-dependent PD-1/PD-L1 checkpoints and immunosuppressive cells cooperate in pathological states since as described above, the activation of the PD-1 protein can enhance the differentiation of immunosuppressive cells, and vice versa, the cytokines secreted by immunosuppressive cells can stimulate the expression of the PD-1/PD-L1 proteins (Figs. 1 and 2).

Intriguingly, many crucial enhancers of the aging process are inducers of PD-L1 expression. Cellular senescence might represent the final outcome since many SASP components are important enhancers of PD-L1 expression, whereas an increased expression of PD-L1 inhibits the immune surveillance of senescent cells. Moreover, many inflammatory SASP factors, such as chemokines and complements, promote the recruitment of immunosuppressive cells into aged tissues, thus enhancing the development of an immunosuppressive microenvironment as well as a more systemic immunosenescence. It is likely that many inflammatory mediators strive to stimulate the PD/PD-L1 pathway in order to prevent excessive inflammatory injuries in tissues. Another interesting observation indicates that a decline in the autophagy stimulates the accumulation of PD-L1 proteins because the turnover of PD-L1 proteins is regulated by selective autophagy. In this situation, the signaling pathways activating mTOR signaling have a crucial role since they can also stimulate the expression of the immunosuppressive PD-L1 protein in non-senescent cells. Many investigators have claimed that mTOR signaling has a key role in the aging process. For example, activation of the insulin/IGF-1 pathway stimulates mTOR signaling and consequently can activate STAT3 signaling which promotes the expression of PD-L1 and enhances immunosuppression [196–198]. There is clear evidence that insulin/IGF-1 signaling can promote the aging process although it has critical functions in protein synthesis and energy metabolism [199]. Interestingly, current anti-aging therapies, i.e., metformin and rapamycin, are inhibitors of mTOR signaling and accordingly potent stimulators of autophagy. It seems that the decline in autophagy not only disturbs the cellular proteostasis and tissue homeostasis but via the activation of PD-L1 signaling, it can enhance the accumulation of senescent cells and thus promote the aging process.

## Conclusions

The presence of a chronic low-grade inflammation with aging remodels the properties of the immune system, inducing a permanent decline in its functional activities, a state called immunosenescence. It is not only during the aging process where one encounters immunosenescence, it also exists in many chronic age-related inflammatory diseases and cancer microenvironment. It is known that a defect in immune surveillance has a key role in allowing tumor cells to evade immune clearance. In tumors, the activation of immunosuppressive cells and many inhibitory immune checkpoints, such as the PD-1/PD-L1 pathway, disturb the function of immune cells, especially that of the cytotoxic CD8^+^ T and NK cells. Interestingly, it was recently demonstrated that the expression of the immunosuppressive PD-L1 protein was robustly upregulated in senescent cells and that this phenomenon suppressed the cytotoxicity of CD8^+^ T cells [20]. This is an important observation since it indicates that senescent cells can be protected against immune surveillance and subsequent clearance, i.e., leading to their accumulation within aged tissues. The PD-1/PD-L1 axis has a crucial role in tumor growth but it seems that it also enhances the aging process. Intriguingly, the signaling pathways associated with the promotion of the aging process are crucial inducers of the expression of the immunosuppressive PD-L1 protein in senescent cells. This seems paradoxical but currently, the exact role of senescent cells is unknown although most likely they are enhancers of the aging process. Currently, there is intense research to develop senolytic drugs aiming to destroy senescent cells, so-called senolytic therapy [200]. The PD-1/PD-L1 checkpoint inhibitor therapy might represent a promising approach, given that it is being clinically exploited in cancer immunotherapy.
